# Efficacy and Safety of Nicorandil in Preventing Contrast-Induced Nephropathy after Elective Percutaneous Coronary Intervention: A Pooled Analysis of 1229 Patients

**DOI:** 10.1155/2020/4527816

**Published:** 2020-09-04

**Authors:** Bin Yi, Shaoyan Mo, Yumei Jiang, Dingwu Yi, Jinwen Luo, Xiang Chen, Jian Rong

**Affiliations:** ^1^Department of Cardiothoracic Surgery, The First Affiliated Hospital, Sun Yat-Sen University, Guangzhou, China; ^2^Department of Extracorporeal Circulation, Heart Center, The First Affiliated Hospital, Sun Yat-Sen University, Key Laboratory on Assisted Circulation, Ministry of Health, Guangzhou, China; ^3^Department of Cardiac Surgery, The Second Xiangya Hospital, Central South University, Changsha, China; ^4^Department of Cardiothoracic Surgery, Hunan Children's Hospital, Changsha, China; ^5^Department of Anesthesiology, The Sixth Affiliated Hospital, Sun Yat-Sen University, Guangzhou, China

## Abstract

**Background:**

Nicorandil in reducing contrast-induced nephropathy (CIN) following elective percutaneous coronary intervention (PCI) is an inconsistent practice. This article aims to evaluate the efficacy and safety of nicorandil in preventing CIN after elective PCI.

**Methods:**

This is a pooled analysis of patients treated with elective PCI. The primary outcome was the incidence of CIN. The secondary outcomes were major adverse events, including mortality, heart failure, recurrent myocardial infarction, stroke, and renal replacement therapy.

**Results:**

A total of 1229 patients were recruited in our study. With statistical significance, nicorandil lowered the risk of CIN (odds ratio = 0.26; 95% confidence interval = 0.16–0.44; *P* < 0.00001; *I*^2^ = 0%) in patients who underwent elective PCI. In addition, no significant differences were observed in the incidence of mortality, heart failure, recurrent myocardial infarction, stroke, and renal replacement therapy between the two groups (*P* > 0.05).

**Conclusions:**

Our article indicated that nicorandil could prevent CIN without increasing the major adverse events. Furthermore, sufficiently powered and randomized clinical studies are still needed in order to determine the role of nicorandil in preventing CIN after elective PCI.

## 1. Introduction

With the continuous development of percutaneous coronary intervention (PCI), contrast-induced nephropathy (CIN), a serious type of kidney injury caused by the application of iodine-containing contrast agent, becomes a common complication in the management of coronary artery disease (CAD) and leads to an increase in morbidity and mortality during the follow-up periods [1]. In this respect, nicorandil, an adenosine triphosphate-sensitive potassium (K_ATP_) channel opener with nitrate, may be an encouraging therapeutic method for patients and clinicians [2]. The cardioprotective effects of nicorandil have been extensively reported [3, 4]; however, the renoprotective potential is less studied, with discrepant conclusions, especially in elective PCI [5–10]. Therefore, this study might be the ﬁrst attempt to systematically analyze the efficacy and safety of nicorandil in preventing CIN after elective PCI.

## 2. Methods

We conducted this meta-analysis in line with the Preferred Reporting Items for Systematic Reviews and Meta-Analyses (PRISMA) guidelines [11].

### 2.1. Literature Search

The PubMed, Web of Science, Cochrane Library, and SinoMed (Chinese database) were retrieved from the inceptions to 30 November 2019. The search terms and/or text words were “percutaneous coronary intervention,” “heart catheterization,” “cardiac catheterization,” “coronary angioplasty,” “coronary stenting,” “coronary balloon,” “coronary rotational atherectomy,” and “nicorandil.” We also reviewed the references mentioned in searched original articles. There were no language limitations during the literatures search.

### 2.2. Selection Criteria and Data Collection

The inclusion criteria were as follows: (1) they were randomized controlled trials; (2) research subjects were all adults (≥18 years old), who were treated with elective PCI; (3) the intervention was nicorandil or not; and (4) the results reported the incidence of CIN in both groups.

The following studies were excluded: (1) the participants who only underwent CAG or emergent PCI, instead of elective PCI; (2) studies that included children participants; (3) studies that contained dubious data; (4) similar studies, redundant, or duplicate publication.

Data extraction, based on the inclusion and exclusion criteria, was performed by two reviewers independently. If discrepancies arose between the reviewers, the third reviewer would assist. The following indices were extracted from each study: the last name of the first author, year of publication, demographic characteristics of participants, the protocol for nicorandil groups, the protocol for control groups, definition of CIN, incidence of CIN, and major adverse events.

### 2.3. Quality Assessment

Quality assessment was performed according to Jadad scale: (1) randomization: grade 0 signifies unused or improper, grade 1 indicates unknown, and grade 2 means pertinent; (2) blinding method: grade 0 signifies improper or unused, grade 1 indicates unknown, and grade 2 means pertinent; (3) withdrawals and dropouts, grade 1 or 0 suggests mentioned or not, grades 3–5 refer to considered high-quality studies, and grades 0–2 refer to low-quality studies [12].

### 2.4. Statistical Analysis

Review Manager 5.3 (Cochrane Collaboration, London, UK) was used for all statistical analyses [13]. According to the inverse variance method [14], odds ratio (OR) with 95% confidence interval (CI) for dichotomous outcomes was calculated [15]. Heterogeneity was measured by *I*^2^ statistic [16]. When *I*^2^ ≥ 50%, which means significant difference, the random effect model was applied. On the contrary, the fixed effect model was applied, when *I*^2^ < 50%, which indicates no significant differences. According to the difference of nicorandil administrations, the subgroup analysis was conducted. The sensitivity analysis was performed by changing the statistical method and analysis model. A funnel plot graph was used to present the publication bias [17].

## 3. Results

### 3.1. Search Results

**Figure 1:**
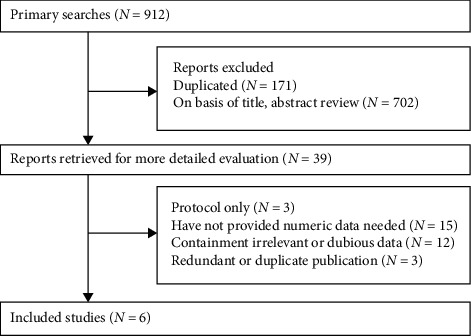
Flowchart of study selection.

A total of 1229 participants who underwent elective PCI were included in six randomized controlled trials [5–10].  Figure 1 indicates the flow chart of literature retrieval. Four studies were from China [5–7, 9], and the remaining studies were from Japan [10] and Iran [8], respectively. The contrast media used in all trials were iodine-based, such as iomeprol [10], iohexol [5, 8, 10], ioversol [6], and Ultravist [7, 9]. All participants in nicorandil groups were treated with nicorandil; however, the route of nicorandil administration applied in these studies differed: oral administration was applied in three studies [6, 8, 9], and intravenous administration was applied in the others [5, 7, 10]. In addition, participants in control groups were treated with matching placebo. The main characteristics and quality assessments of these studies are detailed in Table 1.

### 3.2. Comparison of CIN

**Figure 2:**
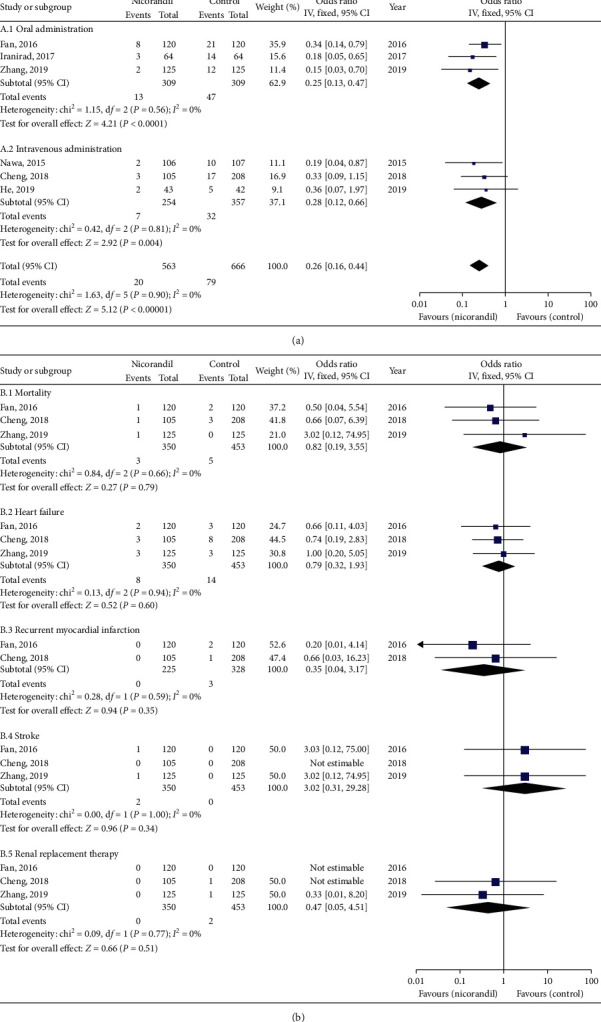
Comparison of nicorandil versus control group for the incidence of (a) contrast-induced nephropathy (CIN) and (b) major adverse events. Abbreviations: IV, inverse variance method; CI, confidence interval.

**Figure 3:**
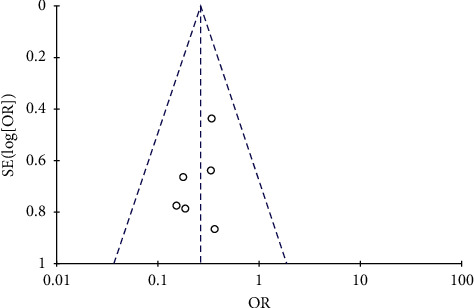
Funnel plot of standard error by log odds ratio. Abbreviations: SE, standard error; OR, odds ratio.

All included six studies described the incidence of CIN [5–10]. Given *I*^2^ = 0%, which means no significant differences, the fix effect model was applied. Compared with the control group, the patients who underwent elective PCI had a significantly lower CIN incidence in the nicorandil group (six trials; 1229 participants; OR = 0.26; 95% CI = 0.16–0.44; *P* < 0.00001; Figure 2(a)). The sensitivity analysis proved that our analysis was robust by changing the statistical method and analysis model. The nicorandil treatment effect on CIN was not significantly altered, when the fixed effect model was transformed into the random effect model (OR, 0.26; 95% CI = 0.16–0.44). According to the funnel plot of the standard error by log OR, there was no significant publication bias in this meta-analysis (Figure 3).

### 3.3. Subgroup Analysis

It is important to notice that the present result should be regarded with caution because different modes of nicorandil administration were applied in included studies: nicorandil was given orally in three studies and given intravenously in the others. Therefore, we performed a subgroup analysis according to oral administration and intravenous administration, to evaluate the efficacy of nicorandil for CIN. Nicorandil significantly reduced the CIN through oral administration (three trials; 618 participants; OR = 0.25; 95% CI = 0.13–0.47; *P* < 0.0001; *I*^2^ = 0%; Figure 2(a)) [6, 8, 9] and intravenous administration (three trials; 611 patients; OR = 0.28; 95% CI = 0.12–0.66; *P*=0.004; *I*^2^ = 0%; Figure 2(a)) [5, 7, 10].

### 3.4. Comparison of Major Adverse Events

Data about mortality, heart failure, recurrent myocardial infarction, stroke, and renal replacement therapy were available in three trials, and recurrent myocardial infarctions were available in two trials. There was no significant difference between the groups in mortality (OR = 0.82, 95% CI = 0.19–3.55, *P*=0.79, *I*^2^ = 0%) [6, 7, 9], heart failure (OR = 0.79, 95% CI = 0.32–1.93, *P*=0.60, *I*^2^ = 0%) [6, 7, 9], recurrent myocardial infarction (OR = 0.35, 95% CI = 0.04–3.17, *P*=0.35, *I*^2^ = 0%) [7, 9], stroke (OR = 3.02, 95% CI = 0.31–29.28, *P*=0.34, *I*^2^ = 0%) [6, 7, 9], and renal replacement therapy (OR = 0.47, 95% CI = 0.05–4.51, *P*=0.51, *I*^2^ = 0%) [6, 7, 9] after elective PCI (Figure 2(b)).

## 4. Discussion

This meta-analysis found that nicorandil could reduce the incidence of CIN without increasing major adverse events after elective PCI. Moreover, whether taken orally or intravenously, the efficacy of nicorandil in lowering the risk of CIN is not affected.

CIN, which follows stent restenosis and stent thrombosis, is the third major complication after PCI [18]. Previous studies have reported that the incidence of CIN was 3%, and more than 50% of patients experienced CIN for high-risk individuals following PCI [19–21]. Although the mechanism is not yet clear, however, it is believed that the complication is closely related to renal hemodynamic changes, toxic injury of renal tubular epithelial cells, and decrease in nitric oxide production, intracellular calcium overload, and oxidative stress [22–24]. According to the different pathogenesis, many therapies on preventing CIN have emerged. Intravenous volume expansion, which helps maintain patient hemodynamic stability, could exert a renoprotective effect by maintaining renal perfusion. On the other hand, intravenous volume expansion contributes to dilution of contrast agent and other nephrotoxicants, such as reactive oxygen species and cell necrosis factors. Therefore, hydration, the effective way of intravenous volume expansion, is a universally accepted practice to prevent CIN and has been used in all selected trials in this meta-analysis [25–27]. Nicorandil, a nitrate ester compound with K_ATP_ channel opener, could lower the risk of CIN following elective PCI in our study. The possible explanations are as follows. On the one hand, nitrate has the potential to improve the production of nitric oxide in blood vessels, antagonize the generation of intracellular oxygen free radicals, increase the renal blood flow, and relieve the inflammatory reaction [28–30]. On the other hand, the opening of intracellular K_ATP_ channel contributes to promote the hyperpolarization of mitochondrial membrane, inhibit the opening of T-type calcium channel, decrease the contents of oxygen free radicals, and dilate the microvessels [31–33].

All trials included in our analysis applied various usages of nicorandil, and the modes of nicorandil administration included oral administration and intravenous administration. Therefore, to further investigate whether the modes of administration might be an influencing factor to the efficacy of nicorandil, the subgroup analysis was performed according to oral administration and intravenous administration. These results demonstrated a consistent effect of nicorandil on CIN after elective PCI whether oral or intravenous. However, on account of the limitation of the included studies and the small sample size, the finding should be approached with prudence.

Furthermore, our study also assessed the safety of nicorandil. And between both groups, there were no signiﬁcant differences in mortality, heart failure, recurrent myocardial infarction, stroke, and renal replacement therapy, indicating that nicorandil did not raise the risk of major adverse events. However, it is worth noting that a limited amount of data might stop us from identifying a difference.

There are several limitations in our meta-analysis. Firstly, the deﬁnition of CIN is based on a change in serum creatinine; however, serum creatinine values are susceptible to a variety of factors including food, age, and weight [34, 35]. Therefore, the sensitivity of this biomarker is poor in evaluating early renal impairment. Cystatin C, another biomarker that is only cleared in the kidney, which increases when renal function is slightly impaired, is relatively stable for the reason that it is impervious to age, gender, diet, medications, or inflammation [36, 37]. However, the raw data relevant to the continuous variables were a barrier to assess the postoperative renal biomarkers by meta-analyses. Cystatin C could be used to evaluate the CIN following elective PCI in the future study. Secondly, as mentioned above, all trials included in our analysis applied various usages of nicorandil; however, no data were available enough to conduct the further analysis according to nicorandil doses and dosage forms. Moreover, the results should be treated cautiously although the subgroup analysis was performed on the basis of nicorandil administration routes. Thirdly, all patients in enrolled trials were Asians, and the occurrence of the CIN after elective PCI might be related to the region and race; therefore, we still should use caution when drawing conclusions. Finally, the baseline renal functions varied in the recruited patients of all trials, which may bring about the difference in the effect of nicorandil on the CIN; however, we lacked relevant information to make a subgroup.

## 5. Conclusions

All in all, this meta-analysis demonstrates that nicorandil contributes to a decline in the incidence CIN after elective PCI without increasing the major adverse events. However, because of the small sample size and the deficient information of the enrolled patients, the conclusions should be further validated in future well-designed, large-scale, clinical trials.

## Figures and Tables

**Table 1:** Characteristics of each included trials.

Study	No. of patients^a^	Country	Contrast agent	Nicorandil administration	Jadad
Nawa [15]	106/107	Japan	Iomeprol or iohexol	Intravenous administration	4
Fan [14]	120/120	China	Ultravist	Oral administration	4
Iranirad [13]	64/64	Iran	Iohexol	Oral administration	4
Cheng [12]	105/208	China	Ultravist	Intravenous administration	4
He [11]	43/42	China	Iohexol	Intravenous administration	3
Zhang [10]	125/125	China	Ioversol	Oral administration	5

^a^The values are represented as nicorandil group/control group.

## Data Availability

All the data used in the analysis are presented within the manuscript.
